# The Association between Mid-Upper Arm Circumference and Blood Pressure in an Italian Population of School-Aged Children and Adolescents with Lipid Disorders

**DOI:** 10.3390/jcm13030663

**Published:** 2024-01-24

**Authors:** Francesco Martino, Tarcisio Niglio, Francesco Barillà, Eliana Martino, Vincenzo Paravati, Pier Paolo Bassareo

**Affiliations:** 1 Department of Internal Medicine, Anaesthesiology, and Cardiovascular Science, La Sapienza University, 00161 Rome, Italy; elianamartino@inwind.it (E.M.); vincenzo.paravati@uniroma1.it (V.P.); 2 Istituto Superiore di Sanità, 00161 Rome, Italy; posta@tarcisioniglio.it; 3 Department of Systems Medicine, Tor Vergata University, 00133 Rome, Italy; francesco.barilla@uniroma2.it; 4 School of Medicine, University College of Dublin, DO4 W6F6 Dublin, Ireland; piercard@inwind.it; 5 Department of Cardiology, Mater Misericordiae University Hospital, D07 R2WY Dublin, Ireland; 6 Children’s Health Ireland at Crumlin, D12 N512 Dublin, Ireland

**Keywords:** mid-upper arm circumference, body mass index, blood pressure, children, adolescents

## Abstract

**Background:** Many anthropometric measurements have been investigated concerning their association with blood pressure (BP) in paediatric age groups. This study aims to find a relationship between mid-upper arm circumference (MUAC) and BP in a population of children and adolescents aged 1–18 years. **Methods:** 5853 subjects (2977 females and 2876 males) were studied. MUAC, body mass index (BMI), and BP were measured. The individuals in the study were subdivided and grouped by gender and type of school attended in Italy: 1–5 years (pre-school), 6–10 years (primary school), 11–13 years (secondary school), 14–18 years (high school). **Results:** In the age range of 6–13 years, all the subjects with MUAC > 50th percentile had systolic and diastolic BP significantly higher than children with MUAC below 50th percentile (*p* < 0.0001). In the age range 14–18 years, the relationship persisted only in females (*p* < 0.001 and *p* < 0.05 for diastolic and systolic BP, respectively). A linear relationship was found between MUAC and BMI. **Conclusions:** In Italian children of both genders aged 6–13, arm distribution of body fat is strongly associated with increased systolic and diastolic BP. As such, a simple anthropometric measurement like MUAC might represent a tool to identify young subjects who are at risk for HTN.

## 1. Introduction

Arterial hypertension (HTN) is considered one of the main and potentially reversible causes of cardiovascular diseases (CVD) and death worldwide [1,2]. In addition, HTN is a risk factor for stroke, chronic renal disease, and vascular dementia [3,4]. The estimated prevalence of HTN in children is 3–5%. It increases up to 10–11% after puberty, thus representing a significant burden [5,6]. A recent meta-analysis of 47 studies carried out over a period of 25 years (1990–2004) confirmed that the overall prevalence of HTN in children is 4% [5]. Other more recent studies conducted in Australia, Canada, and central Europe reported a disease prevalence of 3.1%, 4.5%, and 6%, respectively [7,8,9]. In Italy, the overall prevalence is 4.1% and increases with weight. In fact, it is 1.4% in children with normal weight but rises to 7.1% in those who are overweight and to 25% in those who are obese [10]. In babies, secondary HTN is the most diffuse form of sustained high blood pressure (BP). However, circumstances have changed over the last two to three decades, and the prevalence of primary HTN has shown an upward trend after the age of 6 [11].

The American Heart Association and American Academy of Paediatrics have highlighted the importance of primary prevention in reducing the risk of CVD in young people. Unfortunately, paediatric HTN is a significant risk factor for developing early atherosclerosis [12,13]. Therefore, starting primary prevention at an early age is crucial [14,15]. Given this context, regular BP checks are recommended from childhood, according to the suggestions provided by the American Academy of Paediatrics as well as the European Society of Cardiology [16,17]. The current guidelines recommend at least three BP checks/year after the age of 3. Multiple BP measurements are needed before classifying a child as hypertensive. After diagnosing HTN in a child, secondary high BP should be ruled out [16,17,18].

Overweight and obesity are independent risk factors in the development of HTN from infancy [19,20]. In fact, paediatric HTN is quite widespread among overweight and obese children [21,22]. This is particularly evident in ethnic minorities as testified by the Houston Screening Project [23]. Both paediatric HTN and obesity prevalences are growing, and there is a direct relationship between BP and body mass index (BMI). In some studies, the prevalence of HTN is about 30% in obese children [24]. In a large retrospective study involving 801,019 children and adolescents aged 3–17, a normal–high weight (that is, a BMI over the 60th percentile by age) was associated with an increased risk of developing HTN. Not only that is true, but the higher the BMI, the higher the risk [25]. In a Canadian study, after measuring BP at 12.9, 15.2, and 17 years, each unit increase in BMI was associated with an increase in systolic BP equal to 0.7 mmHg [26]. BMI, waist circumference (WC), waist-to-height ratio (WHtR), and waist-to-flank ratio are anthropometric measurements related to the onset of paediatric HTN [27]. Among them, BMI and WC are the best in predicting the occurrence of high BP [28,29]. However, other studies demonstrated that, since BMI is essentially linked with abdominal fat distribution, whereas BP increases the most with height, BMI may underestimate the risk of HTN in short stature children and overestimate the same risk in tall subjects [30,31,32,33,34]. Because of such an inconsistency, new anthropometric measurements have been suggested as predictors of HTN in childhood, such as a body shape index (ABSI), body roundness index (BRI), and waist-by-hip ratio. All of them have proven to be linked with the occurrence of HTN, metabolic syndrome, and cardio-metabolic risk factors (CMRF) [35,36,37]. In addition, the association between ABSI and height does not depend on BMI [38,39]. Other studies carried out in Asia showed that WHR is superior to BMI and WC in predicting the onset of HTN in adolescence [35]. Nevertheless, other studies have questioned this point [40]. Also, neck circumference (NC), wrist circumference (WrC), WHtR, and waist-by-hip ratio are able to identify CMRF in the paediatric as well as adult populations [41,42,43,44].

While the association between BMI and raised BP has been well known for a long time, lean body mass (LBM) might also be associated with abnormal BP in young adults who are not overweight or obese. In fact, LBM estimated by mid-upper arm muscle circumference (MAMC) seems to be correlated with cardiac mass and stroke volume, which in turn strongly influence BP [45,46]. MAMC is calculated from mid-upper arm circumference (MUAC) minus triceps skinfold thickness [47,48,49]. At present there are very few studies on MUAC in cardiology as well [47,50].

This study aims to check if MUAC is able to predict HTN in a population of Italian children and adolescents.

## 2. Materials and Methods

The present study was carried out by analysing a sample size of children and adolescents undergoing examinations at the Lipid Clinic Research Centre (Department of Paediatrics, Sapienza University of Rome, Rome, Italy) to confirm the presence of abnormal lipid values previously detected during an ordinary laboratory test. The data were gathered over the period of 1995–2011.

Out of over 50,000 subjects who were examined, in only 5853 of them were all the required demographic (age below 18 years), anatomical (height, weight, MUAC) and physiological (heart rate, systolic and diastolic BP) features required to be enrolled in the study available. A significant percentage of them had high cholesterol levels. Subjects with secondary HTN as well as those taking medications for BP control were excluded from the study.

### 2.1. Mid-Upper Arm Circumference and Anthropometric Measurements

MUAC was measured to the nearest 0.1 cm using a non-elastic flexible tape in the standing position with abdomen relaxed, arms at their sides, and feet together. It was measured at the mid-point between the tip of the shoulder and the tip of the elbow of the left arm [51].

Body weight was measured in light clothes and without shoes and was approximated to the nearest 0.1 kg on a mobile digital scale (Seca, Hamburg, Germany). Height was measured to the nearest 0.1 cm using a wall mounted stadiometer (Seca, Hamburg, Germany). BMI was calculated in each participant as weight (kilograms) over height (meters squared).

At least three consecutive measurements for all anthropometric variables were taken for each child, and their mean was used for analysis.

### 2.2. Blood Pressure and Other Measurements

BP was measured using a mercury sphygmomanometer, with an appropriate cuff for each child’s upper arm size; three cuffs with different bladder sizes (8 × 13, 9 × 23, and 12 × 22 cm) were used [52].

Systolic BP was defined by the onset of the first Korotkoff sound and diastolic BP was indicated by the fifth Korotkoff sound (disappearance of Korotkoff sound). BP measurement was approximated to the nearest 2 mmHg. BP was measured in the clinic by the same physician on the same occasion as the anthropometric measurements, while children were sitting and with the cubital fossa supported at heart level, after at least 5 min of rest. Three measurements were taken over a given period of 5 min and their average was used for calculation [53].

### 2.3. Ethics

Each enrolled child’s legal guardian signed a written informed consent document in accordance with EU Regulation n.679/2016.

This is an observational and retrospective scientific work. It was performed by using the clinical records of children and adolescents examined in Rome, Italy, at Policlinico Umberto I over a period of about fifteen years. Italian law (see D.Lgs. n.211/2003 on Gazzetta Ufficiale of Repubblica Italiana n.184 9 August 2003—supplement to “serie generale”) does not require the authorization of the Ethics Committee in cases of epidemiological and observational studies. The authors used the available clinical records and did not perform BP and anthropometric measurements themselves.

### 2.4. Statistics

All data were collected into a Microsoft Access 2017 database and were analysed by Epi-Info 7 programs (CDC and NIH, 2021 Italian version 7.2.5.0). Statistical analysis estimated descriptive statistics, frequencies, and significance of group differences. Statistical significance between and within groups was calculated based on continuous variables. An analysis of variance (ANOVA) was performed to test the equality of means between groups for continuous variables, including Bonferroni, Kruskal–Wallis, and Newman–Keuls pairwise mean comparison tests. A Chi-square Yates’ correction test was used for non-continuous variables in StatCalc and Analysis programs. The odds ratio was calculated as well. A *p* level less than 0.05 was considered significant.

## 3. Results

This study was performed on a sample of 5853 children (2977 females and 2876 males). The percentage of dyslipidemic patients was 61.6% (1914 females and 1682 males). Anthropometric measurements (height, weight, body mass index (BMI), MUAC, and heart rate) and their relationships are shown in Table 1, Table 2, Table 3 and Table 4 and stratified by gender and age.

Table 5 reports mean diastolic BP and Table 3 shows mean systolic BP stratified by gender, age, and MUAC. We used the MUAC 50th percentile to divide all children (stratified by gender and age) into two further groups: those with MUAC below 50th percentile and those with MUAC over 50th percentile.

In regards to MUAC, the 50th percentile was used as a cut-off simply because in our sample size, all the subjects with MUAC > 50th percentile had a BMI that was significantly higher than children with MUAC below 50th percentile (see Table 4 as well). These differences were statistically significant.

Table 5 and Table 6 show mean diastolic and systolic BP grouped by gender, sex, and type of school attended in Italy: 1–5 years (pre-school), 6–10 years (primary school), 11–13 years (secondary school), 14–18 years (high school). All children aged 6–13 showed statistically significant differences in diastolic and systolic BP after stratification by MUAC 50th percentile. In the 14–18 years age range, the relationship persisted only in females.

The results illustrated in Table 7 and Table 8 show an odds ratio between 1.9 and 2.8 thus demonstrating, in our sample, an association between BP and MUAC. At the same time, the 95% confidence interval does not include the value 1, which means that the results are statistically significant.

## 4. Discussion

Cardiovascular diseases (CVD) are the leading cause of early death in the US and the rest of the world [54]. In Italy, they are responsible for 34.81% of all deaths [55]. CVDs are multifactorial. Some of the risk factors cannot be changed (age, gender, and genetics), while others are modifiable (such as diet, alcohol consumption, and smoking habits). In particular, the latter may contribute to cause both HTN and overweight/obesity [56,57,58]. HTN in children and adolescents is related to the development of CVD in adulthood. For example, Daniels et al. demonstrated that 8% of hypertensive children and adolescents have already developed an increase in their cardiac mass, which is a known risk factor for developing CVD [59]. HTN, even in paediatric age, is often asymptomatic; therefore, the early identification of the subjects who have high BP plays a pivotal role in terms of primary prevention [60,61,62]. This is with a goal of preventing the onset of CVD later on. In fact, CVDs are the consequence of a chronic degenerative inflammatory process which starts in the perinatal period and infancy [15,63,64,65]. Therefore, BP screening in paediatric age groups is crucial in attempting to reduce the global CVD risk burden [14].

MUAC is often used in assessing nutritional status [66]. It is also an important marker of obesity in children [67,68,69]. Several studies have found that MUAC is linked with BP in paediatric age groups. For example, in the Brompton study, BP was linked with MUAC at all ages studied [70]. Larger MUAC at 1 year of age predicted higher systolic BP at 6 years [71].

The results of this study show that both systolic and diastolic BP are significantly influenced by MUAC in the age range 6–13 years. This is consistent with the results outlined by Ma et al. In fact, MUAC was a simple, cheap, and accurate tool for identifying children of Chinese Han ethnicity aged 7–12 years with HTN. MUAC was equivalent in accuracy to BMI and WC as a screening test for HTN [72]. This is confirmed in Italian children of both genders as well. In children aged 1–5 years, the relationship is lost, whereas in those aged 14–18, it is weaker and persists just in females. This is probably due to the fact that in MUAC, tissue composition is different between the two genders. MUAC is a combination of fat and muscle mass. The proportion of muscle mass is greater in males and increases with time, and this is why the relationship between MUAC and BP is lost in adolescent males. Conversely, it persists, although attenuated, in females. The novelty of this study is that the association between MUAC and BP is confirmed even in a population of different ethnicity and with hypercholesterolaemia.

MUAC in turn has a direct relationship with BMI. As such, MUAC has been suggested as an alternative tool in screening overweight and obesity in paediatric age groups [73,74,75].

The association between juvenile overweight/obesity and HTN is well known [76,77]. It is multifactorial and involves fluid and sodium retention, insulin resistance, the sympathic nervous system activation, the renin–angiotensin–aldosterone axis stimulation, and other mechanisms like adipokines release and endothelial dysfunction [78]. Regarding the first mechanism (e.g., fluid and sodium retention), human and mammalian kidneys are embedded in adipose tissue. This in turn can enter the renal hilum and medulla, thus reducing blood flow, causing tubular compression, and increasing sodium and fluid absorption [79]. Concerning insulin resistance, it leads to fluid and sodium retention and vasoconstriction, thus increasing BP [80,81]. Weight loss is associated with a decrease in BP and an increase in insulin sensitivity [82]. Children with borderline BP had higher weight and insulin blood levels compared with their normotensive peers [83]. Again, insulin resistance can increase BP through the renin–angiotensin–aldosterone system activation as well as acting through the central nervous system [84]. An imbalance in the link between the sympathetic nervous system, heart, arterial vessels, and renin–angiotensin–aldosterone system leads to HTN [85]. Fasting supresses the sympathetic nervous system activation, whereas an excessive carbohydrates and lipids intake activates it [86]. The renin–angiotensin–aldosterone system is involved in regulating the tone and sodium absorption of efferent glomerular arterioles on the basis of salt intake and BP levels. A system imbalance can alter natriuresis and BP control [78]. Increased plasmatic renin activity leads to an increase in aldosterone activity and BP which is higher in obese adolescents than in their counterparts with normal weight. Furthermore, weight loss in obese adolescents is related to a significant decrease in aldosterone levels and BP levels [87]. Another mechanism is inflammation with the release of adipokines which causes marked vasoconstriction and increased BP [88,89,90]. Again, an excess in body fat causes endothelial dysfunction and reduces nitric oxide release, thus favouring the development of HTN [91,92,93].

The authors performed quite a similar study in 2013. However, the 2013 paper was conducted on a different and smaller sample size (n = 370 vs. n = 5835). A more advanced statistical analysis was performed in the present study. The 2013 results showed that MUAC was significantly higher in hypertensives than normotensives. Systolic BP was correlated with MUAC which in turn was strongly related with BMI [56].

The present study has its strengths and limitations. The strengths include the large sample size and complex statistics applied to confirm the association between MUAC and systolic and diastolic BP. As for the limitations, the statistical analysis used showed no cause–effect relationship between MUAC and BP. Neither an ANOVA nor an odds ratio allow for the determination of causation or direct influence but rather show difference between groups and likelihood/odds of an outcome, respectively. There is a positive association between MUAC and BMI as well as a non-consequential correlation between MUAC and BP. This can be used as a simple method for diagnosis even by somebody who is not a doctor. In other words, high BP can be suspected simply by measuring MUAC with a dressmaker’s tape. Of course, the definite diagnosis of HTN remains a clinician’s responsibility. Furthermore, since a significant percentage of the subjects in the study had high cholesterol levels, the results of this study may not be extended to other populations.

## 5. Conclusions

In conclusion, in Italian children of both genders aged 6–13 and with lipid disorders, peripheral distribution of body fat is associated with increased systolic and diastolic BP. As such, a simple anthropometric measurement like MUAC might represent a tool to identify young subjects who are at risk of HTN [94].

## Figures and Tables

**Table 1:** Patients’ height and weight.

F + M		Female			Male		
Age (y)	Pt (n)	Pt (n)	Height Mean ± SD (cm)	Weight Mean ± SD (kg)	Pt (n)	Height Mean ± SD (cm)	Weight Mean ± SD (kg)
1	7	4	---	---	3	---	---
2	57	34	---	---	23	---	---
3	135	75	82 ± 7	10 ± 2	60	82 ± 6	11 ± 2
4	201	109	94 ± 2	13 ± 1	92	95 ± 2	14 ± 1
5	238	108	103 ± 3	16 ± 1	130	104 ± 3	17 ± 3
6	259	140	116 ± 13	23 ± 11	119	112 ± 7	21 ± 4
7	485	267	120 ± 8	24 ± 7	218	122 ± 7	25 ± 6
8	611	302	126 ± 7	28 ± 7	309	127 ± 7	28 ± 6
9	689	346	130 ± 8	30 ± 8	343	131 ± 8	31 ± 8
10	774	385	136 ± 9	35 ± 11	389	137 ± 9	37 ± 12
11	511	248	145 ± 8	40 ± 10	263	145 ± 7	42 ± 12
12	605	280	151 ± 7	46 ± 10	325	149 ± 9	45 ± 12
13	541	279	156 ± 10	51 ± 13	262	155 ± 11	50 ± 13
14	385	207	158 ± 8	55 ± 13	178	162 ± 8	59 ± 12
15	147	78	164 ± 10	64 ± 17	69	168 ± 8	63 ± 15
16	116	73	170 ± 10	68 ± 17	43	176 ± 9	80 ± 26
17	59	23	177 ± 9	74 ± 18	36	176 ± 6	80 ± 27
18	33	19	183 ± 3	68 ± 2	14	177 ± 5	67 ± 7
total	5853	2977			2876		

Acronyms: F = female; M = male; y = years; Pt = patients; n = number; cm = centimetres; kg = kilograms; SD = standard deviation; --- = not reported.

**Table 2:** Patients’ BMI and heart rate.

F + M		Female			Male		
Age (y)	Pt (n)	Pt (n)	BMI Mean ± SD (Ratio)	Heart Rate Mean ± SD (rpm)	Pt (n)	BMI Mean ± SD (Ratio)	Heart Rate Mean ± SD (bpm)
1	7	4	---	---	3	---	---
2	57	34	---	---	23	---	---
3	135	75	16.0 ± 1	103 ± 11	60	16.2 ± 2	106 ± 22
4	201	109	16.1 ± 3	99 ± 13	92	15.8 ± 2	98 ± 11
5	238	108	16.3 ± 2	93 ± 15	130	16.6 ± 2	93 ± 12
6	259	140	16.9 ± 3	86 ± 12	119	16.8 ± 2	84 ± 8
7	485	267	17.1 ± 3	83 ± 10	218	17.4 ± 3	81 ± 9
8	611	302	17.7 ± 3	84 ± 10	309	17.8 ± 3	81 ± 10
9	689	346	17.9 ± 3	82 ± 10	343	18.2 ± 4	81 ± 10
10	774	385	19.0 ± 4	80 ± 10	389	19.6 ± 5	79 ± 10
11	511	248	18.8 ± 3	78 ± 10	263	20.5 ± 4	77 ± 9
12	605	280	20.0 ± 3	78 ± 8	325	20.5 ± 4	76 ± 9
13	541	279	21.0 ± 4	75 ± 9	262	20.6 ± 4	76 ± 8
14	385	207	21.6 ± 4	76 ± 8	178	22.4 ± 4	76 ± 9
15	147	78	23.3 ± 5	76 ± 7	69	22.1 ± 4	69 ± 7
16	116	73	22.7 ± 3	73 ± 12	43	24.2 ± 5	71 ± 10
17	59	23	22.2 ± 4	67 ± 11	36	24.1 ± 6	69 ± 8
18	33	19	24.5 ± 6	61 ± 15	14	22.0 ± 3	66 ± 9
total	5853	2977			2876		

Acronyms: F = female; M = male; Pt = patients; n = number; bpm = beats per minute; SD = standard deviation; --- = not reported.

**Table 3:** Anthropometric measurements.

F + M		Female			Male		
Age (y)	Pt (n)	Pt (n)	BMI Mean ± SD (Ratio)	MUAC Mean ± SD (cm)	Pt (n)	BMI Mean ± SD (Ratio)	MUAC Mean ± SD (cm)
1	7	4	---	---	3	---	---
2	57	34	---	---	23	---	---
3	135	75	16.0 ± 1	16 ± 1	60	16.2 ± 2	15 ± 3
4	201	109	16.1 ± 3	17 ± 1	92	15.8 ± 2	15 ± 2
5	238	108	16.3 ± 2	18 ± 2	130	16.6 ± 2	18 ± 2
6	259	140	16.9 ± 3	19 ± 2	119	16.8 ± 2	18 ± 2
7	485	267	17.1 ± 3	18 ± 3	218	17.4 ± 3	19 ± 3
8	611	302	17.7 ± 3	19 ± 2	309	17.8 ± 3	20 ± 2
9	689	346	17.9 ± 3	20 ± 3	343	18.2 ± 4	22 ± 3
10	774	385	19.0 ± 4	21 ± 3	389	19.6 ± 5	21 ± 3
11	511	248	18.8 ± 3	21 ± 3	263	20.5 ± 4	23 ± 5
12	605	280	20.0 ± 3	23 ± 4	325	20.5 ± 4	23 ± 3
13	541	279	21.0 ± 4	24 ± 3	262	20.6 ± 4	24 ± 3
14	385	207	21.6 ± 4	24 ± 6	178	22.4 ± 4	25 ± 3
15	147	78	23.3 ± 5	28 ± 5	69	22.1 ± 4	25 ± 4
16	116	73	22.7 ± 3	25 ± 2	43	24.2 ± 5	28 ± 4
17	59	23	22.2 ± 4	27 ± 3	36	24.1 ± 6	29 ± 6
18	33	19	24.5 ± 6	27 ± 4	14	22.0 ± 3	29 ± 2
total	5853	2977			2876		

Acronyms: F = female; M = male; Pt = patients; n = number; y = years; cm = centimetres; SD = standard deviation; --- = not reported.

**Table 4:** Relationship between MUAC and BMI.

Age [y] Pt (n)	Gender	MUAC 50th Centile	Pt (n)	BMI	SD	*p*
[1,5] (110)	F	below	28	15	2	<0.0001 ^(1)^
		over	24	18	3	
	M	below	33	15	1	<0.001 ^(2)^
		over	25	17	2	
	F + M	below	61	15	1	<0.0001 ^(3)^
		over	49	18	3	
[6,9] (1135)	F	below	301	15	2	<0.0001 ^(4)^
		over	229	21	3	
	M	below	327	16	3	<0.0001 ^(5)^
		over	278	22	4	
	F + M	below	628	15	3	<0.0001 ^(6)^
		over	507	21	4	
[10,12] (757)	F	below	207	17	2	<0.0001 ^(7)^
		over	151	23	3	
	M	below	199	17	2	<0.0001 ^(8)^
		over	200	23	3	
	F + M	below	406	17	2	<0.0001 ^(9)^
		over	351	23	3	
[13,17] (295)	F	below	95	20	3	<0.0001 ^(10)^
		over	59	26	6	
	M	below	65	19	3	< 0.0001 ^(11)^
		over	76	25	4	
	F + M	below	160	19	3	<0.0001 ^(12)^
		over	135	25	5	

Acronyms: F = female; M = male; y = years; Pt = patients; n = number; SD = standard deviation; --- = not reported. ^(1)^ *p* < 0.0001 (Kruskal–Wallis H = 22.4390 with df = 1), ^(2)^ *p* = 0.0003 (Kruskal–Wallis H = 13.3006 with df = 1), ^(3)^ *p* < 0.0001 (Kruskal–Wallis H = 36.4810 with df = 1), ^(4)^ *p* < 0.0001 (Kruskal–Wallis H = 292.8857 with df = 1), ^(5)^ *p* < 0.0001 (Kruskal–Wallis H = 352.0538 with df = 1), ^(6)^ *p* < 0.0001 (Kruskal–Wallis H = 644.6958 with df = 1), ^(7)^ *p* < 0.0001 (Kruskal–Wallis H = 184.9815 with df = 1), ^(8)^ *p* < 0.0001 (Kruskal–Wallis H = 228.8176 with df = 1), ^(9)^ *p* < 0.0001 (Kruskal–Wallis H = 418.7230 with df = 1), ^(10)^ *p* < 0.0001 (Kruskal–Wallis H = 66.3119 with df = 1), ^(11)^ *p* < 0.0001 (Kruskal–Wallis H = 22.4390 with df = 1), ^(12)^ *p* < 0.0001 (Kruskal–Wallis H = 139.8159 with df = 1). Mean systolic and diastolic BP were different when stratified by gender and age. All these differences were statistically significant with *p* < 0.001.

**Table 5:** Relationship between MUAC and diastolic BP.

Age [y] Pt (n)	Gender	MUAC	Patients (n)	Mean DBP	Std. Dev.	*p*
[1,5] (110)	F	below	28	61	9	n.s.
		over	24	62	11	
	M	below	33	60	6	n.s.
		over	25	62	6	
	F + M	below	61	61	7	n.s.
		over	49	62	8	
[6,9] (1135)	F	below	301	60	9	<0.0001 ^(1)^
		over	229	64	10	
	M	below	327	60	9	<0.0001 ^(2)^
		over	278	64	9	
	F + M	below	628	60	9	<0.0001 ^(3)^
		over	507	64	10	
[10,12] (757)	F	below	207	60	10	<0.001 ^(4)^
		over	151	65	10	
	M	below	199	61	8	<0.0001 ^(5)^
		over	200	65	12	
	F + M	below	406	61	9	<0.0001 ^(6)^
		over	351	65	11	
[13,17] (295)	F	below	95	62	9	<0.001 ^(7)^
		over	59	68	10	
	M	below	65	66	9	n.s.
		over	76	65	10	
	F + M	below	160	63	9	trend ^(8)^
		over	135	66	10	

Acronyms: F = female; M = male; y = years; Pt = patients; n = number; SD = standard deviation; DBP = diastolic blood pressure; n.s. = not statistically significant. ^(1)^ *p* < 0.0001 (Kruskal–Wallis H = 23.8709 with df = 1), ^(2)^ *p* < 0.0001 (Kruskal–Wallis H = 17.4307 with df = 1), ^(3)^ *p* < 0.0001 (Kruskal–Wallis H = 40.9695 with df = 1), ^(4)^ *p* = 0.0003 (Kruskal–Wallis H = 12.8884 with df = 1), ^(5)^ *p* = 0.0003 (Kruskal–Wallis H = 13.0276 with df = 1), ^(6)^ *p* < 0.0001 (Kruskal–Wallis H = 26.6238 with df = 1), ^(7)^ *p* = 0.0009 (Kruskal–Wallis H = 10.9229 with df = 1), ^(8)^ *p* = 0.0535 (Kruskal–Wallis H = 3.7272 with df = 1).

**Table 6:** Relationship between MUAC and systolic BP.

Age [y] Pt (n)	Gender	MUAC	Patients (n)	Mean SBP	Std. Dev.	*p*
[1,5] (110)	F	below	28	99	12	n.s.
		over	24	100	12	
	M	below	33	97	9	n.s.
		over	25	99	10	
	F + M	below	61	98	10	n.s.
		over	49	99	10	
[6,9] (1135)	F	below	301	96	10	<0.0001 ^(9)^
		over	229	103	10	
	M	below	327	96	9	<0.0001 ^(10)^
		over	278	104	10	
	F + M	below	628	96	9	<0.0001 ^(11)^
		over	507	103	10	
[10,12] (757)	F	below	207	99	10	<0.0001 ^(12)^
		over	151	109	10	
	M	below	199	102	9	<0.0001 ^(13)^
		over	200	111	11	
	F + M	below	406	101	9	<0.0001 ^(14)^
		over	351	110	11	
[13,17] (295)	F	below	95	106	10	<0.05 ^(15)^
		over	59	112	13	
	M	below	65	112	11	n.s.
		over	76	113	13	
	F + M	below	160	108	11	<0.05 ^(16)^
		over	135	113	13	

Acronyms: F = female; M = male; y = years; Pt = patients; n = number; SD = standard deviation; SBP = systolic blood pressure; n.s. = not statistically significant. ^(9)^ *p* < 0.0001 (Kruskal–Wallis H = 68.3079 with df = 1), ^(10)^ *p* < 0.0001 (Kruskal–Wallis H = 71.1723 with df = 1), ^(11)^ *p* < 0.0001 (Kruskal–Wallis H = 139.0866 with df = 1), ^(12)^ *p* < 0.0001 (Kruskal–Wallis H = 63.1113 with df = 1), ^(13)^ *p* < 0.0001 (Kruskal–Wallis H = 55.9281 with df = 1), ^(14)^ *p* < 0.0001 (Kruskal–Wallis H = 122.8109 with df = 1), ^(15)^ *p* = 0.0225 (Kruskal–Wallis H = 5.2053 with df = 1), ^(16)^ *p* = 0.0101 (Kruskal–Wallis H = 6.6194 with df = 1).

**Table 7:** The odds ratio of the children’s frequencies when making a comparison between diastolic BP (50th percentile) and MUAC (50th percentile).

	MUAC 50th		
DBP	Below	Over	Total
Below	904	587	1491
Row%	60.63%	39.37%	100.00%
Col%	72.03%	56.33%	64.91%
Over	351	455	806
Row%	43.55%	56.45%	100.00%
Col%	27.97%	43.67%	35.09%
Total	1255	1042	2297
Row%	54.64%	45.36%	100.00%
Col%	100.00%	100.00%	100.00%
	**Point**	**95% Confidence Interval**	
	**Estimate**	**Lower**	**Upper**
PARAMETERS: Odds-based			
Odds Ratio (cross product)	1.9963	1.6780	2.3751 (T)
Odds Ratio (MLE)	1.9957	1.6778	2.3753 (M)
		1.6715	2.3843 (F)
PARAMETERS: Risk-based			
Risk Ratio (RR)	1.3923	1.2742	1.5213 (T)
Risk Difference (RD%)	17.0821	12.8551	21.3090 (T)
STATISTICAL TESTS	Chi-square	1-tailed p	2-tailed p
Chi-square—uncorrected	61.5946		0.0000000000
Chi-square—Mantel–Haenszel	61.5678		0.0000000000
Chi-square—corrected (Yates)	60.9073		0.0000000000
Mid-p exact		0.0000000000	
Fisher exact		0.0000000000	0.0000000000

**Table 8:** The odds ratio of children’s frequencies when making a comparison between systolic BP (50th percentile) and MUAC (50th percentile of sample).

	MUAC 50th		
SBP	Below	Over	Total
Below	950	545	1495
Row%	63.55%	36.45%	100.00%
Col%	75.70%	52.30%	65.08%
Over	305	497	802
Row%	38.03%	61.97%	100.00%
Col%	24.30%	47.70%	34.92%
Total	1255	1042	2297
Row%	54.64%	45.36%	100.00%
Col%	100.00%	100.00%	100.00%
	**Point**	**95% Confidence Interval**	
	**Estimate**	**Lower**	**Upper**
PARAMETERS: Odds-based			
Odds Ratio (cross product)	2.8404	2.3791	3.3913 (T)
Odds Ratio (MLE)	2.8391	2.3789	3.3918 (M)
		2.3698	3.4052 (F)
PARAMETERS: Risk-based			
Risk Ratio (RR)	1.6709	1.5175	1.8399 (T)
Risk Difference (RD%)	25.5152	21.3630	29.6675 (T)
STATISTICAL TESTS	Chi-square	1-tailed p	2-tailed p
Chi-square—uncorrected	137.1084		0.0000000000
Chi-square—Mantel–Haenszel	137.0487		0.0000000000
Chi-square—corrected (Yates)	136.0808		0.0000000000
Mid-p exact		0.0000000000	
Fisher exact		0.0000000000	0.0000000000

## Data Availability

The analysed data are available on request to T.N.
